# Usual Choline Intake of Australian Children 6–24 Months: Findings from the Australian Feeding Infants and Toddlers Study (OzFITS 2021)

**DOI:** 10.3390/nu16121927

**Published:** 2024-06-18

**Authors:** Zhixiao Li, Shao J. Zhou, Tim J. Green, Najma A. Moumin

**Affiliations:** 1 School of Agriculture, Food & Wine, The University of Adelaide, Adelaide, SA 5000, Australia; zhixiao.li@adelaide.edu.au (Z.L.); jo.zhou@adelaide.edu.au (S.J.Z.); 2 Women and Kids Theme, South Australian Health and Medical Research Institute, Adelaide, SA 5000, Australia; tgreen@flinders.edu.au; 3 Robinson Research Institute, The University of Adelaide, Adelaide, SA 5000, Australia; 4 College of Nursing and Health Sciences, Flinders University, Adelaide, SA 5000, Australia; 5 Adelaide Medical School, The University of Adelaide, Adelaide, SA 5000, Australia

**Keywords:** infants, toddlers, choline, dietary intake, Australia

## Abstract

(1) Background: Despite the important role choline plays in child development, there are no data on dietary choline intake in early childhood in Australia. (2) Aim: In this cross-sectional study, we estimated the usual total choline intake and the proportion exceeding the Adequate Intake (AI) and determined the main dietary sources of choline in infants 6–12 months (n = 286) and toddlers 12–24 months (n = 475) of age. (3) Methods: A single 24-h food record with repeats collected during the 2021 Australian Feeding Infants and Toddlers Study (OzFITS 2021) was used to estimate dietary choline intake. (4) Results: The mean choline intake was 142 ± 1.9 mg/day in infants and 181 ± 1.2 mg/day in toddlers. Only 35% of infants and 23% of toddlers exceeded the AI for choline based on Nutrient Reference Values (NRVs) for Australia and New Zealand. Breastmilk was the leading source of choline, contributing 42% and 14% of total choline intake in infants and toddlers, respectively; however, egg consumers had the highest adjusted choline intakes and probability of exceeding the AI. (5) Conclusions: Findings suggest that choline intake may be suboptimal in Australian infants and toddlers. Further research to examine the impact of low choline intake on child development is warranted.

## 1. Introduction

Choline is required for lipid transport, maintenance of the structural integrity of cell membranes, and DNA methylation [1,2]. Most importantly, choline is a precursor for acetylcholine synthesis, a key neurotransmitter involved in learning, memory, and muscle coordination [1,2,3]. Although choline can be synthesized endogenously in the liver, the amount produced is insufficient to meet physiological needs, and the remainder must be sourced from the diet [2,3]. Choline exists in both fat-soluble and water-soluble forms. Animal-source foods such as meat, fish, poultry, eggs, and dairy are higher in choline, particularly the fat-soluble forms phosphatidylcholine and sphingomyelin, whereas plant-based sources and breastmilk are higher in the water-soluble forms free choline, phosphocholine, and glycerophosphocholine [1,4]. 

During periods of rapid growth and development such as the first two years of life, demand for choline is particularly high, ~125–200 mg/day [5,6]. The 2006 Nutrient Reference Values (NRVs) for Australia and New Zealand [5], based on the United States Institute of Medicine Dietary Reference Intakes [6], have established Adequate Intakes (AIs) for different life stage groups, including children under 2 years; however, no studies have investigated dietary choline intakes in young children in Australia. Nutrition surveys conducted in Europe and Canada have shown choline intakes are low, with few children <3 years exceeding the AI [7,8]. However, in the United States National Health and Nutrition Examination Survey 2009–2014 [9], toddlers 2–3 years were more likely than all other age groups to be above the AI. 

In 2021, we published the first-ever Australia-wide food and nutrient intake study in children under two years [10,11], the Australian Feeding Infants and Toddlers Study (OzFITS 2021). Although diets were sufficient for most nutrients, we reported a high prevalence of inadequacy for iron and zinc in infants, 75% and 17%, respectively [11]. Although lower, one quarter of toddlers were also below the estimated average requirement (EAR) for iron. Low consumption of red meat, poultry, and fish contributed to low iron and zinc intake [11,12,13]. Animal-source foods, including eggs and dairy, are important dietary sources of choline; however, food composition values for choline were not available in the 2011–13 Australian Food, Supplement, and Nutrient Database (AUSNUT) [14] at the time of this analysis. Thus, the aims of this study are: (1) to estimate the usual total choline intake of children 6–24 months; (2) to estimate the proportion exceeding the AI; and (3) to determine the major dietary sources contributing to choline intake.

## 2. Materials and Methods

### 2.1. Study Design and Participant Recruitment

Details of the data collection methods used in OzFITS 2021 are available elsewhere [10,13]. Briefly, OzFITS 2021 was a cross-sectional study designed to assess early feeding practices and food and nutrient intake in Australian children 0–24 months. Recruitment began in April 2020 via targeted online advertising [15]; 1140 caregiver–child dyads were enrolled over a 12 month period The Women’s and Children’s Health Network Human Research Ethics Committee (HREC/19/WCHN/44) approved the study. Verbal informed consent was obtained from all caregivers prior to data collection.

### 2.2. Data Collection

Data were recorded and managed using a secure, web-based data management system, Research Electronic Data Capture (REDCap) [16,17]. 

#### 2.2.1. Caregiver Feeding Practices

Caregiver feeding practices pertaining to breastfeeding history, breastmilk substitute use, and age of introduction to complementary foods and common food allergens were captured using a telephone-based questionnaire. 

#### 2.2.2. Dietary Intake Data

Caregivers of exclusively breastfed infants were not asked to complete a 24-h food record, as breastmilk alone is sufficient until ~6 months. All caregivers of children who received breastmilk substitutes and/or commenced complementary feeding were eligible for a 24-h food record. Of these, 30% were also randomly assigned to complete another food record. 

Caregivers recorded all foods, beverages, and dietary supplements consumed by their child from midnight the previous night to midnight the following night on their assigned record-keeping day(s) using the food record booklet and portion estimation guide provided [18,19]. Breastfeeds were recorded as minutes suckling at the breast between two and ten minutes and converted to a volume of intake [20,21,22]. The amount of breastmilk substitute consumed was calculated as follows: dry powder (g)/total prepared volume (mL) × prepared volume consumed (mL).

#### 2.2.3. Dietary Data Entry

Caregivers sent photos or scanned copies of the completed food record booklets via email. Upon receipt, study staff completed a follow-up interview with caregivers to confirm the contents of the food record using the multiple-pass interview method [23]. Data were entered directly into Xyris FoodWorks Professional version 10 [24]. Nutrient values were assigned to foods and beverages using the 2011/13 Australian Food, Supplement, and Nutrient Database (AUSNUT) [14] and a study-specific commercial infant and toddler food database [25]. Food group codes according to the AUSNUT classification system were assigned to foods and recipes [26]. 

Since publication of the original study [11], choline data of foods and breastmilk have become available in Xyris FoodWorks™. Using the updated 2011/13 AUSNUT data source published by Probst et al. [27], we assigned choline values to dietary data collected in OzFITS 2021. In addition, we updated the commercial infant and toddler food database with choline values using the recipe approach as previously described [10,13]. Manufacturers of breastmilk substitutes that did not include choline as a nutrient on the nutrient information panel were contacted and asked to provide estimates of naturally occurring choline per 100 g product using their product composition information.

### 2.3. Data Analysis

Using both days’ dietary intake data, the sum choline from all food, beverages, breastmilk, and breastmilk substitutes was calculated. The distribution of usual choline intake was estimated using the Intake Modelling, Assessment, and Planning Program (IMAPP); intra-individual variation intake was removed using the Iowa State University method [28]. Adjusted choline intakes were compared to the NRVs for Australia and New Zealand [5]. As choline lacks an EAR, the probability of being above the AI was reported for infants and toddlers [5]. Only 6% of caregivers reported using supplements, none of which included choline; therefore, nutrient contribution from supplement use was excluded.

As all caregivers completed at least one day of dietary intake data for their child, classification as a consumer or non-consumer of a food or food group was based on consumption behavior on the first day. The Pearson chi square test of independence was used to assess whether consumption of choline-rich foods influenced the likelihood of exceeding the AI for choline. The level of significance was set at 0.05. Descriptive statistics are reported as mean ± SD or SE, median (IQR), or n (%) as appropriate. All statistical analyses were completed using SPSS Statistics (v29.0, IBM Corp., Armonk, New York, NY, USA).

## 3. Results

### 3.1. Participant Characteristics

A total of 1140 caregiver–child dyads were enrolled in OzFITS 2021; 164 of them were exclusively breastfed and were excluded from the analysis, as they did not complete a food record. The remaining 976 were eligible for a food record and included in the analysis. Of those eligible for a food record, 308 and 542 were caregivers of infants 6–12 months and toddlers 12–24 months, respectively. Attrition was low, with 286/308 (93%) caregivers of infants 6–12 months completing at least one food record and 102/308 (33%) completing a repeat record on a non-consecutive day. Similarly, 475/542 (88%) of caregivers of toddlers 12–24 months completed one food record and 153/542 (28%) completed a second day’s intake. Three quarters of caregivers had at least a bachelor’s degree, and 60% reported household incomes in the top two quintiles. Prevalence of egg allergy was low, with 3% (22/850) of caregivers reporting a confirmed allergy (Table 1). 

### 3.2. Distribution of Usual Choline Intake

The distribution of usual choline intake in infants 6–12 months and toddlers 12–24 months is summarized in Table 2. A total of 761 children who completed the OzFITS 2021 aged 6–24 were included. The proportion of the population above the AI for choline was low in both age groups at 35% and 23%, respectively.

### 3.3. Adjusted Choline Intakes of Consumers and Non-Consumers of Choline-Rich Foods

Table 3 summarizes the proportion of infants and toddlers above the AI for choline and their adjusted choline intakes according to consumption of choline-rich foods. Infants 6–12 months who consumed eggs, fish/seafood, and dairy milk had consistently higher adjusted choline intakes and were more likely to be above the AI for choline compared to non-consumers of these foods. Similarly, toddlers who consumed eggs and fish/seafood had significantly higher median choline intakes, and a greater proportion exceeded the AI compared to non-consumers. Toddlers who consumed formula had both the lowest adjusted choline intakes and probability of being above AI for choline.

### 3.4. Top Sources of Choline

Table 4 illustrates the foods or food groups contributing ≥ 5% of total choline and the percentage of all choline derived from these sources for consumers and in the overall population. In total, 78% (222/286) of infants 6–12 months were breastfed, and breastmilk accounted for almost 60% of all choline among breastfed infants. Eggs were consumed by fewer than one fifth of infants, but provided almost one third of the choline among consumers and was the fourth highest source of choline overall.

Similarly, breastmilk was the leading source of choline among toddlers, comprising one third of all choline intake among consumers. Although 62% (294/475) of toddlers consumed dairy milk either as a main drink or as a component of a mixed dish, the overall contribution to total choline intake was low at 16%. Conversely, eggs were consumed by only 22% (105/475) of toddlers but provided 40% of all choline among consumers. Poultry and meat were consumed by less than one third of toddlers; among consumers, these food groups contributed less than one fifth of all choline and only 6% overall. 

## 4. Discussion

This study builds on previous research conducted by our group assessing nutrient intakes in young children to include choline. Adjusted mean choline intakes in infants 6–12 months and toddlers 12–24 months were lower than the recommended intakes endorsed by Australia’s National Health and Medical Research Council (NHMRC) of 150 and 200 mg/day, respectively [5]. The proportion of infants and toddlers exceeding the AI was also low, at 35% and 23%. In both age groups, breastmilk was the leading source of choline, providing 32–56% of all choline among consumers. Other than breastmilk, egg consumers had the highest adjusted choline intakes and were most likely to exceed the AI. 

The AI for choline during lactation is high, at 550 mg/day, to support both maternal and infant needs [2,5]. Despite this recommendation, mean choline intakes among lactating women in the 2011/13 Australian National Nutrition and Physical Activity Survey (NNPAS) were significantly low, at 253 mg/day, with fewer than 1% exceeding the AI [27]. Maternal diet may influence breastmilk composition for some nutrients [29]; however, the predominant forms of choline found in breastmilk are the water-soluble forms free choline, phosphocholine, and glycerophosphocholine, which do not appear to be associated with maternal choline intake [30,31]. 

A total of 78% (222/286) of infants 6–12 months sourced over half of their daily choline from breastmilk; however, few exceeded the AI, as choline-rich complementary foods such as beef (95 mg/100 g), chicken (117 mg/100 g), fish (68 mg/100 g), and egg (230 mg/100 g) [14] were not frequently consumed. Importantly, foods of animal origin are rich in phosphatidylcholine, the most biologically abundant phospholipid, accounting for ~50% of the phospholipids found on cell membranes [2,32]. Unless acquired from the diet, phosphatidylcholine and sphingomyelin must be synthesized from choline, which is also a precursor for acetylcholine [3]. Low intake of these fat-soluble forms of choline may have implications for the overall supply of choline, potentially compromising the structural integrity of cellular membranes and/or neurodevelopment [33,34]; however, further research is needed to determine the optimal ratio of water-soluble and fat-soluble forms of choline for development. 

Although no published studies have evaluated choline intakes in infants, our toddler intake data are comparable with those reported in Canada and Europe [7,8] and are slightly lower than those of the United States (US) [9]. In a randomized controlled trial with Canadian toddlers aged 1–2 years supplemented with long-chain polyunsaturated fatty acids (n = 110), Weideman et al. reported mean choline intakes of 174 ± 56 and 205 ± 67.5 mg/day at one year and two years of age, respectively [8]. Similarly, mean intakes for toddlers 1 to ≤3 years from national nutrition surveys conducted in Finland, Germany, Italy, and the United Kingdom (UK) ranged from 151–210 mg/day [7]. Intakes did not exceed the AI for most toddlers in Canada and Europe [7,8], consistent with our findings. 

In contrast, Wallace et al. reported a higher mean intake (217 ± 3.58) and percentage of toddlers 2–3 years above the AI (61%) in the US NHANES 2009–2014 [9]. This difference may be attributable to the age of the children at the time of the survey and differences in food consumption patterns, particularly meat and dairy foods. Indeed, in the 2016 US Feeding Infants and Toddlers Study, Roess et al. reported that ~70% of toddlers aged 1–2 years consumed meat, poultry, or fish, and 83% consumed cow’s milk as their main drink [35]. In contrast, only 20–27% of toddlers in our study consumed these foods, and 44% received breastmilk as their main milk. After 12 months, the NHMRC Infant Feeding Guidelines recommend cow’s milk as a main drink and continued breastfeeding to two years and beyond; breastmilk substitutes are not necessary for otherwise healthy toddlers [36]. Although breastfeeding is encouraged in the second year, average breastmilk intake for breastfed toddlers was nearly twice the amount of cow’s milk consumed by toddlers who received cow’s milk as a main drink (~340 vs. 209 mL/day) [13] and may have displaced consumption of nutritious foods consistent with the Australian Guide to Health Eating [37]. Compared to their non-breastfed counterparts, breastfed toddlers consumed fewer servings of fruit, vegetables, cereals and grains, and dairy foods [13]. Furthermore, breastmilk substitutes (toddler milks), which are not necessary, were consumed by one fifth of toddlers and, unlike infant formula [38], do not contain added choline. Caregivers may benefit from additional guidance on how to prioritize consumption of animal-source foods rich in choline and other essential nutrients in the second year while continuing to breastfeed and limit breastmilk substitute use. 

Australia is the world capital for food allergies, with 1/10 infants diagnosed with an IgE-mediated food allergy by 12 months [39]. To mitigate this, the Australasian Society for Clinical Immunology and Allergy (ASCIA) published new guidelines for the prevention of food allergy in 2016, recommending the introduction to common food allergens before 12 months [40]. To maintain/develop tolerance to these foods, ASCIA further recommends regular consumption (twice weekly) [41]. Compliance with the timing of introduction was high, with 95% of OzFITS 2021 participants receiving hen’s egg by 12 months [42]; however, the frequency of egg consumption per week in this population is unknown, as our dietary survey was limited to two days. Only 2.6% (22/850) of caregivers reported a confirmed egg allergy; therefore, it is unlikely this contributed to the overall low prevalence of egg consumption and suggests eggs may be infrequently consumed.

A key strength of this study is that it is the first to provide data on the distribution of usual choline intake in infants aged 6–12 months, as well as dietary sources contributing to choline intake. It also adds to the limited body of evidence and provides contemporary intake data from a cohort of Australian toddlers aged 12–24 months. Nevertheless, our study did have some limitations. Although the United States Institute of Medicine Dietary Reference Intakes, on which the NRVs for Australia and New Zealand are based, identified choline as an essential nutrient in 1998 [6], an estimated average requirement (EAR) has yet to be established for this nutrient due to limited experimental evidence. As such, AIs based on the median intakes of healthy individuals in a population group are used to describe choline intakes [5]. While intakes above the AI can be assumed to be sufficient, intakes below the AI cannot be used to infer risk of inadequacy [5,6]. For older infants aged 7–12 months, the AI is based on the average concentration of choline in breastmilk and adjusted for body size and does not account for choline derived from complementary foods [5,6]. Similarly, the AI for toddlers aged 1–3 years is not based on population intakes; rather, it is extrapolated from adults, with adjustments made for body size and growth. Choline from breastmilk may have been under- or overestimated, as the volume equations used to calculate breastmilk intake apply a constant flow rate and do not account for differences in feeding efficiency between children [43]. Finally, caregivers may have underreported unhealthy foods due to social desirability bias; however, energy intakes in our study were on average 10% higher than the estimated energy requirement, and 9/10 toddlers consumed discretionary foods higher in saturated fat, added sugar, or salt [11,13,44] on the day of the survey, suggesting this was not a problem. Finally, we did not assess choline status, as this would require a blood draw, and caregivers in our study were not willing to subject their young children to blood collection. In addition, assessment of choline status is challenging [45].

## 5. Conclusions

Average choline intakes among Australian infants and toddlers were below recommendations, and fewer than one third were above the AI. Breastmilk was the leading source of choline in both age groups; however, egg consumers had the highest adjusted choline intakes and probability of exceeding the AI. Caregivers should aim to include a serving of animal-source foods such as egg, fish/seafood, poultry or red meat into the diets of their young children to improve choline intake. Further research to examine the impact of low choline intake on child development is warranted. 

## Figures and Tables

**Table 1:** Participant characteristics of caregiver–child dyads (n = 850).

Characteristic	Mean (SD) or n (%)
*Caregiver*	
Age, years	34 ± 4.6
Education	
Year 10 or 11	7 (1)
Secondary school	40 (5)
Certificate or diploma	167 (20)
Bachelor’s degree or above	636 (75)
Annual household income (AUD)	
<40,000	38 (5)
40,001–70,000	95 (11)
70,001–105,000	191 (23)
105,001–205,000	404 (48)
>205,000	110 (13)
Prefer not to disclose	12 (1)
*Child*	
Age, months	
6–12 months	8.5 ± 1.7
12–24 months	17.7 ± 3.3
Sex (female)	
6–12 months	152 (49)
12–24 months	252 (47)
Confirmed egg allergy	
6–12 months	7 (2)
12–24 months	15 (3)

**Table 2:** Percentiles of choline intake and the proportion of Australian children 6–24 months (n = 761) above the Adequate Intake and Upper Limit ^1^.

	NRVs		Distribution of Choline Intake						NRV Compliance (%)	
	AI ^2^	UL ^2^	10th	25th	50th	Mean ± SE	75th	90th	>AI	>UL
Infants 6–12 months										
Choline, mg/day	150	–	103	121	139	142 ± 1.9	159	187	35	–
Toddlers 12–24 months										
Choline, mg/day	200	1000	147	161	179	181 ± 1.2	198	218	23	0

^1^ Choline intake data are presented as percentiles of usual intake, mean ± SE, or percentages of NRV. NRVs, nutrient reference values; AI, adequate intake, UL, tolerable upper level of intake; ^2^ National Health and Medical Research Council Nutrient Reference Values for Australia and New Zealand [5].

**Table 3:** Percentiles of choline intake and the percentage of children 6–24 months (n = 761) above the Adequate Intake consuming or not consuming choline-rich foods ^1^.

	Consumers			Non-Consumers			*p*
	n (%)	Choline (mg/d) 50th (25th, 75th)	% > AI ^2^	n (%)	Choline (mg/d) 50th (25th, 75th)	% > AI ^2^	
***Infants 6–12 months* (n = 286)**							
Breastmilk	222 (78)	139 (124, 159)	38	64 (22)	134 (114, 149)	25	0.058
Breastmilk substitutes	106 (37)	138 (121, 166)	32	180 (63)	138 (121,159)	37	0.432
Meat	62 (22)	144 (131, 175)	45	224 (78)	137 (119,157)	32	0.057
Poultry	60 (21)	145 (127, 170)	42	226 (79)	137 (120,156)	33	0.221
Fish/Seafood	41 (14)	152 (124, 171)	54	245 (86)	138 (120, 155)	32	0.007
Eggs	49 (17)	159 (141, 194)	65	237 (83)	119 (152, 237)	29	<0.001
Milk	63 (22)	146 (128, 175)	49	223 (78)	138 (120, 155)	31	0.007
Yoghurt	118 (41)	142 (127, 173)	44	168 (59)	135 (119, 152)	29	0.007
***Toddlers 12–24 months* (n = 475)**							
Breastmilk	209 (44)	182 (169, 199)	25	266 (56)	177 (157,197)	22	0.430
Breastmilk substitutes	89 (19)	175 (157, 193)	16	386 (81)	180 (163, 199)	25	0.065
Meat	129 (27)	180 (167, 200)	26	346 (73)	179 (160, 198)	22	0.445
Poultry	144 (30)	181 (169, 201)	27	331 (70)	179 (160, 196)	21	0.181
Fish/Seafood	97 (20)	187 (170, 204)	31	378 (80)	177 (159, 196)	21	0.042
Eggs	105 (22)	202 (187, 222)	53	370 (78)	174 (158, 189)	15	<0.001
Milk	294 (62)	180 (161, 199)	25	181 (38)	178 (162, 196)	20	0.271
Yoghurt	253 (53)	179 (162, 196)	22	222 (47)	180 (160, 199)	25	0.434

^1^ Adjusted choline intakes obtained using the Intake Modelling Assessment and Planning Program (IMAPP); ^2^ AI, adequate intake. The AI for choline for infants 6–12 months and toddlers 12–24 months is 150 mg/d and 200 mg/d, respectively [5].

**Table 4:** Sources of choline by food or food group contributing ≥ 5% choline on day one of the food record in Australian children 6–24 months (n = 761).

Food or Food Group	Consumers n (%)	Choline Intake (mg/d) among Consumers 50th (25th, 75th)	Contribution to Choline Intake (%) among Consumers 50th (25th, 75th)	Overall Percentage of Total Choline Intake for All Participants
***Infants 6–12 months* (n = 286)**				
Breastmilk	222 (78)	78 (53, 103)	56 (36, 77)	42
Breastmilk substitutes	106 (37)	58 (28, 79)	44 (19, 62)	14
Vegetables	236 (83)	8 (3, 18)	6 (3, 12)	8
Eggs	49 (17)	61 (19, 89)	28 (15, 41)	7
***Toddlers 12–24 months* (n = 475)**				
Breastmilk	209 (44)	54 (32, 86)	32 (17, 47)	14
Dairy milk	294 (62)	26 (14, 44)	16 (7, 27)	12
Eggs	105 (22)	94 (62, 118)	39 (26, 48)	12
Vegetables	391 (82)	10 (4, 21)	6 (3, 12)	7
Cereal-based dishes	171 (36)	21 (10, 40)	12 (5, 23)	6
Poultry	144 (30)	28 (15,45)	15 (8, 25)	6
Meat	129 (27)	30 (15, 49)	16 (9, 26)	6
Fruit	449 (95)	9 (5,15)	5 (3, 9)	5

## Data Availability

Data are available upon written request to the corresponding author.
